# Effect of High-Pressure Torsion on Phase Formation and Mechanical Properties of a High-Entropy TiZrHfMoCrCo Alloy

**DOI:** 10.3390/ma16247558

**Published:** 2023-12-08

**Authors:** Alena S. Gornakova, Dilara B. Kabirova, Anna Korneva, Boris Straumal, Marcel F. Imayev, Alexei Kuzmin, Paweł Czaja, Natalia S. Afonikova, Valeriy I. Orlov, Alexei N. Nekrasov, Nafis F. Khayretdinov, Gregory Davdian

**Affiliations:** 1 Osipyan Institute of Solid State Physics of the Russian Academy of Sciences, Ac. Osipyan Str. 2, 142432 Chernogolovka, Russia; alenahas@issp.ac.ru (A.S.G.); natasha@issp.ac.ru (N.S.A.); orlov@issp.ac.ru (V.I.O.); faberest@yandex.ru (G.D.); 2 Institute for Metals Superplasticity Problems of Russian Academy of Sciences, Stepan Khalturin Str. 39, 450001 Ufa, Russia; dilara@imsp.ru (D.B.K.); marcel@imsp.ru (M.F.I.); nafis@imsp.ru (N.F.K.); 3 Institute of Metallurgy and Materials Science Polish Academy of Sciences, Reymonta Str. 25, 30-059 Cracow, Poland; a.korniewa@imim.pl (A.K.); p.czaja@imim.pl (P.C.); 4 Department of Physical Chemistry, National University of Science and Technology “MISIS”, Leninsky Avenue 4, 119991 Moscow, Russia; 5 Institute of Solid State Physics, University of Latvia, Kengaraga Str. 8, LV-1063 Riga, Latvia; a.kuzmin@cfi.lu.lv; 6 Korzhinskii Institute of Experimental Mineralogy of the Russian Academy of Sciences, Ac. Osipyan Str. 4, 142432 Chernogolovka, Russia; alex@iem.ac.ru

**Keywords:** high-entropy alloy, EBSD, EXAFS, crystal lattice, phase transformations, three-point bending, microhardness

## Abstract

This investigation delved into the alterations in the mechanical properties of a TiZrHfMoCrCo high-entropy alloy due to phase transformations induced by high-pressure torsion (HPT). The alloy’s genesis involved levitation melting within an argon atmosphere, presenting two distinct states for analysis: the initial, post-manufacturing state and the state subsequent to HPT treatment. The original alloy featured a composition comprising a singular A2 phase with a bcc lattice and two Laves phases, C15 and C14. The HPT process triggered significant phase modifications: a retention of one C15 Laves phase and decomposition of the bcc phase into two distinct phases exhibiting different bcc lattice parameters. The HPT-induced effect prominently manifests as strong grain refinement. However, scanning electron microscopy (SEM) observations unveiled persistent inhomogeneities at a micron scale both before and after HPT treatment. Thus, grain refinement occurs separately within each of the bcc and Laves phases, visible in the light, dark, and gray areas in SEM images, while mixing does not occur on the scale of several microns. The examination of Ti, Cr, Co, Zr, Mo, and Hf via X-ray absorption spectroscopy (EXAFS) at specific K-edges and L3-edge revealed that the HPT treatment conserves the local atomic environment of metal atoms, albeit with a slight elevation in static disorder. Assessments through microhardness and three-point bending tests demonstrated the material’s inherent hardness and brittleness. The microhardness, standing at a substantial value of 600 HV, displayed negligible augmentation post-HPT. However, the microhardness of individual phases exhibited a notable alteration, nearly doubling in magnitude.

## 1. Introduction

Since their inception by Brian Cantor [1], Jian-Wei Ye [2], and collaborators, high-entropy alloys (HEAs), also known as multiprincipal or alloys without a main component have garnered escalating interest. The diverse amalgamation of components within HEAs presents a vast landscape for creating myriad modifications and exploring multifaceted applications. Notably, one avenue in HEA advancement is their utilization in medical implants. The evolution of a novel generation of metallic biomaterials capable of offering both biocompatibility and commendable mechanical properties stands as a pivotal requirement for future medical applications. A recently developed biomaterial by Nagase et al. [3] has captured attention due to its potential as an implant material; however, its publication lacked comprehensive elucidation regarding its mechanical properties [3]. Despite the authors conducting a comprehensive theoretical review and affirming the promise of this HEA in the medical domain, their focus on biocompatibility overshadowed mechanical attributes in their study. Although the biocompatibility analysis of the molten gold ingots containing bio-HEA TiZrHfCr0.2Mo and TiZrHfCo0.07Cr0.07Mo displayed promising results [3], there remains a critical need for further exploration through microstructural and mechanical investigations. Our ongoing research endeavors aim to supplement and broaden the scope of [3], presenting an opportunity to enhance the material or expand its potential applications in this domain.

It is widely recognized that the mechanical characteristics of metallic materials can be fundamentally altered through severe plastic deformation (SPD). This process induces a notable elevation in the density of lattice defects alongside a substantial reduction in grain sizes. Consequently, these changes impart a significant boost to the material’s strength. Among the spectrum of SPD methods available, high-pressure torsion (HPT) [4] stands out as one of the most effective techniques for diminishing grain size. Particularly in metal alloys, HPT tends to facilitate the formation of a nanocrystalline microstructure, thereby leading to a notable enhancement in microhardness [5,6,7,8]. The mechanical properties of a material are intricately tied to its microstructure and phase composition. Therefore, recognizing this close nexus, we have chosen to delve deeper into understanding this relationship within the realm of high-entropy alloys (HEA).

One of the most intricate and informative techniques for investigating the structural aspects of metals and alloys, including HEAs, is electron backscattering diffraction (EBSD) [9]. This method serves to gather crystallographic data concerning a material, encompassing details such as phase distribution, individual grain orientation, and the mutual misorientation of grains, among other characteristics. An EBSD image is generated based on Kikuchi diffraction patterns acquired via a scanning electron microscope (SEM). Describing the structure of HEAs using the EBSD method poses a novel and non-trivial challenge owing to the presence of five or more components, which introduces lattice distortions compared to pure elements. In studies by Wang [10,11], the EBSD technique was applied to analyze average grain size and orientation within Al-CoCrCuFeNi and CoCrCuNiAl alloys. The presented inverse pole figure (IPF) maps revealed challenges in assessing the predominant grain orientation, despite both alloys exhibiting a face-centered cubic (fcc) lattice in their matrix. In the AlCoCrCuFeNi alloy, the <111> grain orientation was predominant, whereas the CoCrCuNiAl alloy displayed a predominance of the <101> orientation. Employing EBSD following various processing methods of an equimolar AlCrFeCoNi alloy, Löbel et al. [12] identified the optimal processing technique by controlling phase distribution, thereby gaining a deeper understanding of microstructural effects. Sun et al. [13] presented a particularly intriguing result by varying the aluminum content (x = 0, 0.3, 0.5, 1) in Al_x_CoCrFeNi HEAs. The IPF maps showcased not only changes in grain size but also a shift in the preferential grain orientation from <011> in the Al0CoCrFeNi alloy to <111> in the Al1CoCrFeNi alloy. These findings underscore the dynamic relationship between grain characteristics and alloy composition within HEAs.

We faced a second challenge in conducting mechanical tests on HEAs. Across numerous studies focusing on HEAs, the standard practice typically involves measuring micro-hardness and/or nanohardness to enable the calculation of Young’s modulus [5,6,7,8,14]. However, the publication of classical tension, compression, or bending tests performed on HEAs remains scarce, being likely attributed to the reduced sample sizes resulting from HPT treatments [15,16].

This study aimed to elucidate the mechanical properties of the promising TiZrHfMoCrCo HEA by extending beyond microhardness measurements to incorporate three-point bending tests, an alternative yet universally recognized method. The comprehensive characterization of mechanical properties was complemented by a thorough examination of the HEA’s microstructure through scanning (SEM) and transmission (TEM) electron microscopy, including EBSD. Additionally, the investigation delved into the HEA’s atomic structure and phase composition through the application of X-ray absorption spectroscopy (EXAFS) and X-ray diffraction (XRD), respectively.

## 2. Materials and Methods

The six-component high-entropy alloy Ti10.8-Zr18.7-Hf42.3-Mo20.7-Cr3.8-Co3.5 (wt.%) or Ti22.1-Zr20.2-Hf23.3-Mo21.4-Cr7.1-Co5.6 (at.%) (subsequent designations in the article TiZrHfMoCrCo) was chosen as the material for this study. To prepare the alloy, the following pure metals were used: titanium grade TI-1 (99.98 wt.% purified with iodide Arkel method), zirconium (99.98 wt.% purified with iodide Arkel method), molybdenum (99.97 wt.%), hafnium (99.95 wt.% purified with iodide Arkel method), cobalt (99.9 wt.%), and chromium (99.99 wt.%). The pure metals were delivered by Special Metallurgy Ltd., Moscow, Russia. The alloy blank in the form of pressed shavings of pure metals was melted through a levitation method conducted within a pure argon atmosphere at an argon pressure of 35–45 kPa in a suspended state with a rotating electromagnetic field frequency of about 200 kHz. Argon was let into the chamber after it was evacuated to 7 × 10^–5^ Pa. After 15–20 s of keeping the molten billet in a liquid state, the field was turned off, and a drop of the alloy fell under the influence of gravity into a copper mold with an internal diameter of about 11 mm, where crystallization occurred [17]. Component analysis was carried out on an FEI dual beam Versa 3D HighVac scanning electron microscope manufactured by FEI (Hillsboro, OR, USA).

The 0.7 mm thick disks were cut from the ingot. Some of the disks were subjected to high-pressure torsion at room temperature for 5 turns of the plunger under a pressure of 7 GPa at a rotation speed of 1 revolution per minute, in a Bridgman anvil-type HPT installation using a computer control manufactured by W. Klement GmbH (Lang, Austria). After HPT, the sample thickness was 0.35 mm. To determine the chemical composition of the samples, a scanning electron microscope (SEM) Tescan Vega TS5130 MM (Tescan Ltd., Brno, Czech Republic) with the INCA Energy 450+ microanalysis system was used, equipped with an Oxford Instruments (Oxford Instruments Ltd., Abingdon, UK) energy dispersion microanalysis prefix. The SEM FEI E-SEM XL30 instrument (manufactured by FEI, Hillsborough, OR, USA) was also used. It was equipped with an EDAX Genesis energy-dispersive X-ray (EDS) spectrometer (FEI, Hillsborough, OR, USA). EBSD analysis was carried out on equipment from Tescan VEGA (Brno, Czech Republic) with an EBSD attachment CHANNEL5. The final polishing of the samples was carried out using a QATM SAPHIR VIBRO vibration installation (QATM GmbH, Mammelzen, Germany). Transmission electron microscopy (TEM) was performed by using a TECNAI G2 FEG super TWIN (200 kV) instrument (manufactured by FEI, Hillsborough, OR, USA) equipped with the EDS spectrometer produced by EDAX (AMETEK, Inc., Berwyn, PA, USA). For the production of TEM thin foils, twin-jet polishing was performed using a D2 electrolyte in a Struers machine (Struers Inc., Cleveland, OH, USA). For the analysis of spot diffraction, TIA version 2.0 software for the Tecnai microscope was applied (FEI, Hillsborough, OR, USA). Additionally, the identification of constituent phases was performed with CARINEV3 version 3.1 software (Cyrille Boudias & Daniel Monceau Ltd., Paris, France). Structural-phase analysis was carried out using a Rigaku SmartLab X-ray diffractometer (Rigaku, Tokyo, Japan) in Cu-K_α1+α2_ radiation, wavelength 0.15419 nm. Phase analysis and calculation of lattice parameters were carried out using the PowderCell 2.4 program (PowderCell for Windows Version 2.4. 03/08/2000, Werner Kraus & Gert Nolze, BAM, Berlin, Germany). Three-point bending tests of HEA samples were carried out on a testing machine for structural materials UTS 111.2-50 (TestSystems Ltd., Ivanovo, Russia) at room temperature. Microhardness measurements of individual structural components of the alloy were carried out using an ITB-1I-MC hardness tester (Metrotest Ltd., Neftekamsk, Russia). The loading lasted 10 s. After testing various loads, the loads of 200 and 10 g were selected for all measurements.

The X-ray absorption spectroscopy experiments were performed at the DESY PETRA-III synchrotron (DESY AG, Hamburg, Germany) using an experimental setup of the P65 Applied XAFS beamline [18]. The monochromatic X-ray beam was obtained using a fixed-exit double-crystal Si(111) monochromator. Harmonic reduction was achieved with two uncoated or Rh-coated silicon plane mirrors. X-ray absorption spectra at the Ti, Cr, Co, Zr, and Mo K-edges and Hf L_3_-edge were measured at room temperature in fluorescence mode. The intensity of the incoming radiation with the energy E was recorded by an ionization chamber (I_0_(E)) located before the sample, whereas the X-ray fluorescence signal from the sample was acquired by a passivated implanted planar silicon (PIPS) detector (I_f_(E)) placed at 90° to the incident beam. The size of the X-ray beam at the sample was equal to 2.5 × 0.3 mm^2^. The X-ray absorption coefficients were calculated as µ(E) = I_f_(E)/I_0_(E). The X-ray absorption near-edge structure (XANES) and extended X-ray absorption fine structure (EXAFS) spectra were extracted from the experimental X-ray absorption spectra using the XAESA code [19] following a conventional procedure [20]. The analysis of the EXAFS contributions from the first coordination shells of five metals was performed within the single-scattering cumulant approximation or the regularization technique [20]. The required backscattering amplitude and phase functions were calculated by the ab initio self-consistent real-space multiple scattering FEFF8.50L code [21,22].

## 3. Results and Discussion

### 3.1. HEA Microstructure in the Initial State

**Figure 1:**
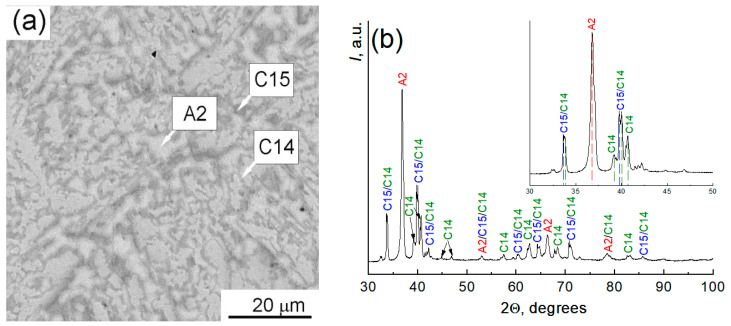
(**a**) SEM image of the as-cast TiZrHfMoCrCo alloy. The bright areas are the A2 phase, the dark areas are the C15 phase, and the gray areas are the C14 phase. (**b**) X-ray diffraction pattern.

Figure 1 presents an SEM image and an X-ray diffraction pattern obtained from the cross-section of a TiZrHfMoCrCo alloy ingot. Examination of the SEM image (Figure 1a) and the diffraction pattern (Figure 1b) revealed the existence of three primary phases within the structure. These phases include the dominant A2 phase, constituting approximately 70% of the volume, alongside two Laves phases, namely C15 and C14. Notably, the intensities of the Bragg peaks corresponding to the two Laves phases, C15 and C14, appear to be roughly equivalent in magnitude.

The base-centered cubic (bcc) phase A2, characterized by a lattice parameter of 0.3448 nm, is visibly brighter in the SEM image (Figure 1a). Conversely, the face-centered cubic (fcc) phase C15 displays a darker appearance and features a lattice parameter of 0.7501 nm. Positioned at the boundaries between the A2 and C15 phases, the hexagonal close-packed (hcp) phase C14 appears light gray and possesses lattice parameters of a = 0.5305 and c = 0.8560 nm. Comprehensive phase parameters and their constituent compositions are outlined in Table 1 for reference and a detailed analysis.

#### 3.1.1. Element Distribution

**Figure 2:**
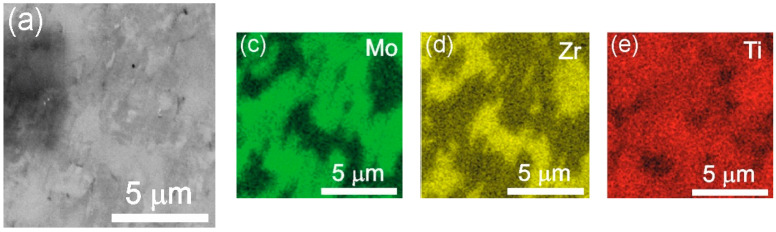

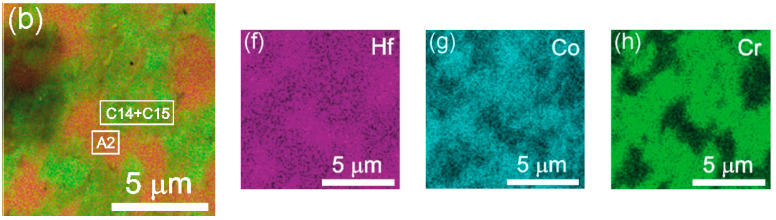
(**a**) SEM image of the as-cast TiZrHfMoCrCo alloy. (**b**) Combined EDS map of elements. (**c**–**h**) EDS element distribution maps for Mo, Zr, Ti, Hf, Co, and Cr.

The TiZrHfMoCrCo HEA comprises six components, indicating a complex arrangement of atoms within its lattice. Given the similarity in atomic sizes, these atoms likely substitute each other at lattice nodes, potentially inducing slight distortions. Elemental distribution maps generated through energy-dispersive X-ray spectroscopy (EDS) were instrumental in establishing the spatial distributions of Mo, Zr, Ti, Hf, Co, and Cr (Figure 2).

After observing the elemental distribution within the alloy, it becomes apparent that hafnium and titanium exhibit a more uniform spread throughout the material compared to other elements. Molybdenum and chromium tend to predominate in certain regions, while zirconium shows prevalence in distinct areas. Cobalt, on the other hand, demonstrates a relatively more even distribution. Specifically, cobalt appears prominently in regions abundant in molybdenum and chromium, but it also manifests in areas rich in zirconium. Our analysis suggests the presence of two primary phases within the investigated TiZrHfMoCrCo alloy: the A2 phase, enriched in Zr, and the C15 phase, exhibiting heightened concentrations of Mo and Cr (Figure 2b). Additionally, a third phase, C14, is notably situated at the boundaries between the A2 and C15 phases, functioning as a transitional or boundary phase.

#### 3.1.2. Component Analysis along a Given Line

**Figure 3:**
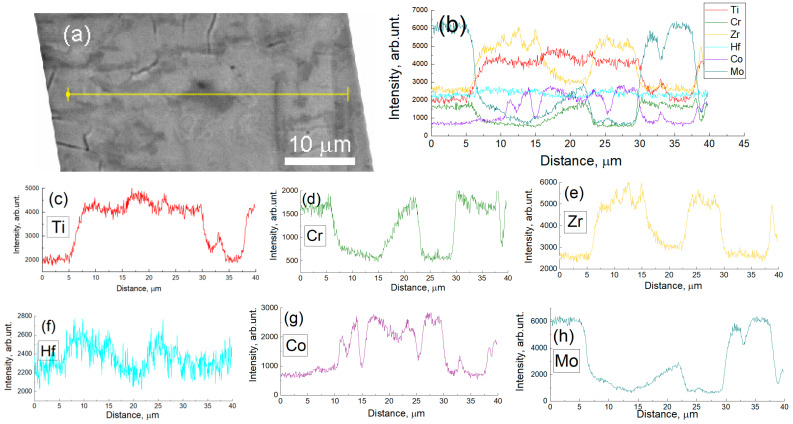
(**a**) SEM image of the TiZrHfMoCrCo alloy with a yellow composition scan line. (**b**) Combined data on the distribution of components along the scan line. (**c**–**h**) Distribution spectra of individual chemical elements.

Figure 3 presents a compositional scan along a linear path, corroborating the findings derived from the elemental distribution maps. Notably, chromium and molybdenum showcase co-localization in specific regions, while zirconium appears in distinct areas. Moreover, cobalt is observed in both chromium and molybdenum-rich regions as well as in zones abundant in zirconium.

#### 3.1.3. EBSD Measurements

**Figure 4:**
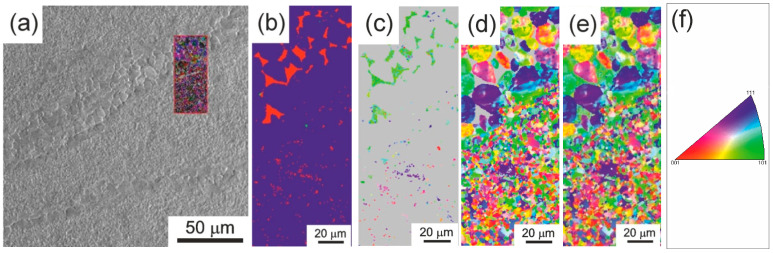
Microstructure of the TiZrHfMoCrCo alloy in its initial as-cast state. (**a**) SEM image of the site from which the EBSD analysis was performed; (**b**) phase distribution: cubic bcc A2 phase (blue), cubic fcc C15 phase (red), and hcp C14 phase (green); (**c**) IPF map of phase C15; (**d**) IPF map of phase A2; (**e**) common IPF map of two phases C15 and A2, (**f**) the color code for the phases to the picture (**d**).

Figure 4 illustrates the outcomes of an EBSD analysis performed on the TiZrHfMoCrCo alloy in its initial as-cast state. In Figure 4b, the predominant phases identified are the bcc A2 phase depicted in blue and the fcc C15 phase represented in red. The sample composition comprises approximately 90 vol. % of the A2 phase, around 9 vol. % of the C15 phase, and approximately 1 vol. % of the C14 phase. Upon revisiting Figure 1b and comparing it with the data obtained from Figure 4b, a discrepancy in the volume fractions of identified phases becomes apparent. Despite both EBSD and XRD methods identifying the same phases, their quantified volume fractions differ. This discrepancy might be attributed to the potential overlap of primary XRD peaks corresponding to the C15 and C14 phases at diffraction angles around 33 and 40°, respectively. In Figure 4b, the fcc phase C15 (depicted in red) is observed along the grain boundaries of the cubic bcc phase A2 (depicted in blue). It is noticeable that certain A2/A2 grain boundaries are entirely infiltrated by layers of the C15 phase, leading to their complete separation. Meanwhile, other A2/A2 grain boundaries exhibit partial wetting by the C15 phase, resulting in the formation of non-zero contact angles between the C15 phase and the A2/A2 boundaries.

**Figure 5:**
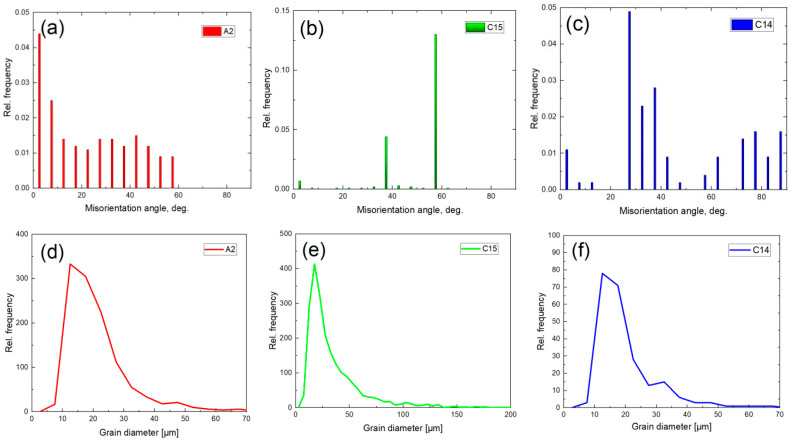
Distributions of misorientation (**a**–**c**) and grain size (**d**–**f**) for each phase in the TiZrHfMoCrCo alloy: (**a**,**d**) A2-phase, (**b**,**e**) C15-phase, and (**c**,**f**) C14-phase.

Processing of EBSD data made it possible to construct the misorientation distributions of grain boundaries (Figure 5a–c) and grain size distributions (Figure 5d–f) in all three phases. Thus, for the C15 phase, only three peaks are observed (Figure 5b). Apparently, this is due to its small volume fraction and, accordingly, to the low number of C15/C15 boundaries.

### 3.2. HEA Microstructure after HPT Treatment

**Figure 6:**
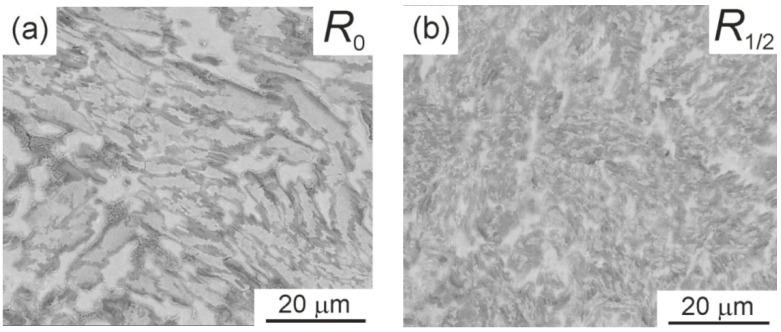

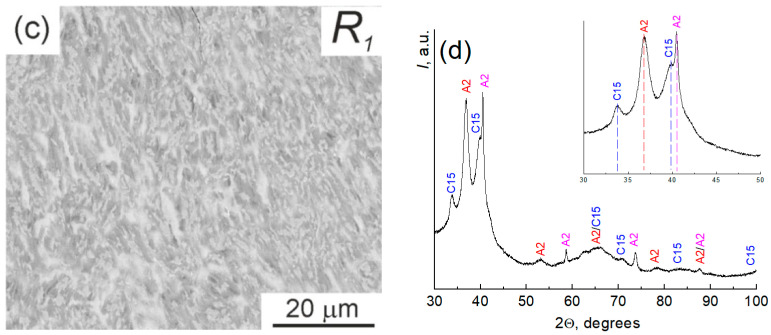
SEM images of the microstructure of the HEA alloy after HPT in three areas: (**a**) *R*_0_ is the central part, (**b**) *R*_1/2_ is the middle of the radius, and (**c**) *R*_1_ is the edge of the sample. (**d**) XRD pattern from the entire surface of the sample.

The microstructure analysis of the HPT-treated sample was conducted using SEM analysis, as depicted in Figure 6. This figure showcases micrographs from three distinct areas of the sample: the central part, denoted as R0 (Figure 6a); the region obtained at the midpoint of the radius R1/2 (Figure 6b); and the sample edge, designated as R1 (Figure 6c). Notably, the discernible size of the regions corresponding to different phases demonstrates a reduction from the sample’s center towards its edge. Moreover, XRD analysis was performed on the entire surface of the sample (Figure 6d). The evaluation revealed the disappearance of the Laves phase C14 post-HPT treatment, while the proportion of the second Laves phase C15 remained relatively unchanged at approximately 25%. Notably, the primary phase A2 underwent a notable transformation, breaking down into two distinct phases, both characterized by an A2 lattice but possessing different lattice parameters.

The A2 phase, indicated by the red dotted vertical line in Figure 6d, corresponds to a hafnium-rich bcc phase with a lattice parameter of 0.3445 nm. Simultaneously, the A2 phase, marked by the pink dotted vertical line in Figure 6d, represents a molybdenum-rich bcc phase with a lattice parameter of 0.3150 nm. The X-ray peaks in Figure 6d exhibit significant broadening in comparison to those observed in the as-cast sample (Figure 1b). This broadening signifies grain refinement within each of the phases as a consequence of the HPT treatment. The microstructures depicted in Figure 6a–c appear strikingly akin to the structure observed in the as-cast sample (Figure 1a) at first glance. Notably, noticeable inhomogeneities at a micron scale persist in these micrographs. This observation implies that grain refinement occurs independently within each phase, evident in the SEM images by variations in light, dark, and gray areas. However, mixing does not manifest at the scale of several microns.

#### Distribution of Elements in the HEA Sample after HPT Treatment

**Figure 7:**
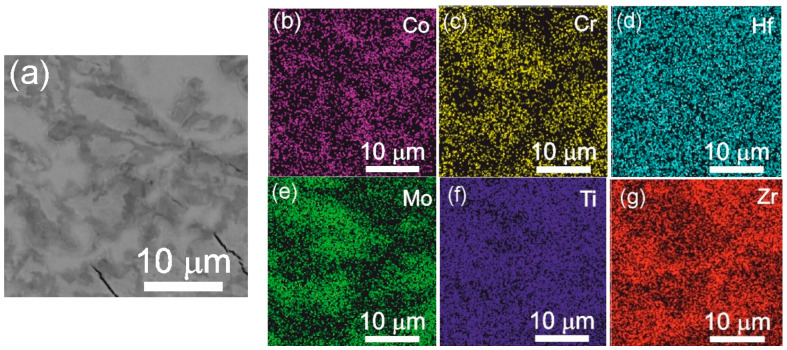
(**a**) SEM image of the TiZrHfMoCrCo alloy after HPT. (**b**–**g**) EDS elemental distribution maps for Co, Cr, Hf, Mo, Ti, and Zr.

A component analysis of the alloy after HPT treatment (Figure 7) showed that in addition to hafnium, which was evenly distributed throughout the sample in the initial state, the treatment led to a uniform distribution of titanium in the alloy. The remaining components form “couples” when distributed in the alloy whereby molybdenum is coupled with chromium and zirconium.

**Figure 8:**
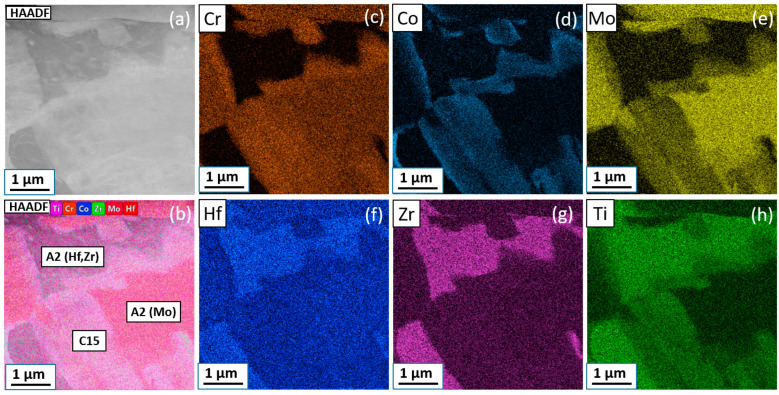
The HAADF/STEM image of the TiZrHfMoCrCo alloy after HPT (**a**), the EDS map of the distribution of all elements in the sample (**b**), and EDS maps of the distribution of elements for Cr, Co, Mo, Hf, Zr, and Ti (**c**–**h**).

Figure 8 depicts the outcomes of the TEM examination conducted on a thin foil of the deformed alloy. In Figure 8a, the microstructure image obtained in scanning microscope mode using a high-angle annular dark field (HAADF) detector is presented. Additionally, Figure 8b showcases an energy-dispersive X-ray spectroscopy (EDS) map highlighting the distribution of all constituent elements, while Figure 8c–h display individual EDS maps revealing the distribution of specific elements—Cr, Co, Mo, Hf, Zr, and Ti, respectively. Analysis of the outcomes in Figure 8b confirms the presence of three distinct phases, aligning closely with the chemical composition observed in the SEM analysis (refer to Table 2). The determination of grain size relied upon a panoramic microstructure image derived from the TEM. Despite the statistical analysis being limited due to a small sample size of approximately 15 grains, it facilitated the estimation of average grain size, which does not exceed 3 µm.

**Figure 9:**
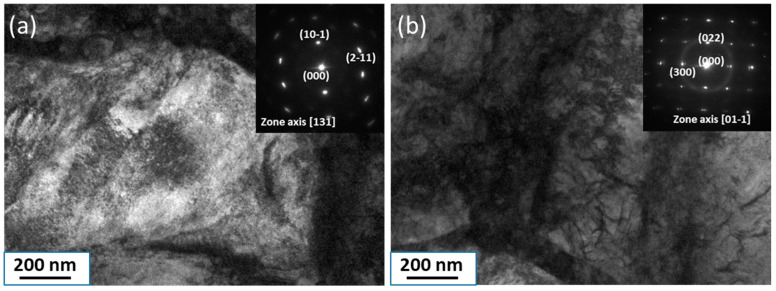
Bright-field TEM micrograph of the A2 (Hf, Zr) phase (**a**) and C15 phase (**b**) with selected electron diffraction patterns observed in the sample after HPT.

Figure 9 shows bright-field TEM micrographs of the internal structure of individual grains of the A2 (Hf, Zr) and C15 phases as examples. A high dislocation density was observed in all phases.

The XRD data reveal a transformation within the as-cast sample, where a single phase displaying the A2 structure undergoes a division into two distinct phases, both retaining the A2 structure but differing in their enrichment levels—one enriched with hafnium, the other with molybdenum. This suggests a localized mass transfer occurring on a scale of several hundred nanometers induced by the HPT process. Assuming a treatment duration of *t* = 300 s with a mass transfer distance of *L* = 100 nm during HPT, employing the formula for bulk diffusion *L* = (*D*_HPT_ *t*)^0.5^ allows to estimate the bulk diffusion coefficient *D*_HPT_, approximating to *D*_HPT_ = 10^−17^ m/s^2^. Extrapolating bulk self-diffusion coefficients for the alloy’s components to room temperature (*T* = 300 K)—the temperature characteristic of HPT processing—yields the following estimated values: bcc titanium *D*_300K_ = 10^−31^ m/s^2^ [23,24], bcc zirconium *D*_300K_ = 10^−28^ m/s^2^ [25,26,27,28], molybdenum *D*_300K_ = 10^−66^ m/s^2^ [29,30,31], chromium *D*_300K_ = 10^−67^ m/s^2^ [32,33], and cobalt *D*_300K_ = 10^−47^ m/s^2^ [34,35]. Consequently, the effective diffusion coefficient *D*_300K_ = 10^−17^ m/s^2^ indicative of mass transfer under HPT is approximately 11–50 orders of magnitude higher than the coefficients of bulk self-diffusion of our alloy at room temperature. Notably, this estimation of bulk self-diffusion coefficients at room temperature disregards the influence of high pressure, which typically significantly retards kinetic processes in solids [36,37]. This substantial acceleration of mass transfer induced by HPT—observed in previous studies concerning the competition between decomposition and formation of supersaturated solid solutions in various binary alloys—is corroborated in our findings [38,39,40,41,42,43,44,45,46,47]. Additionally, based on Figure 9a, an approximate average grain size estimation ranges from 1 to 3 µm.

### 3.3. X-ray Absorption Spectroscopy

**Figure 10:**
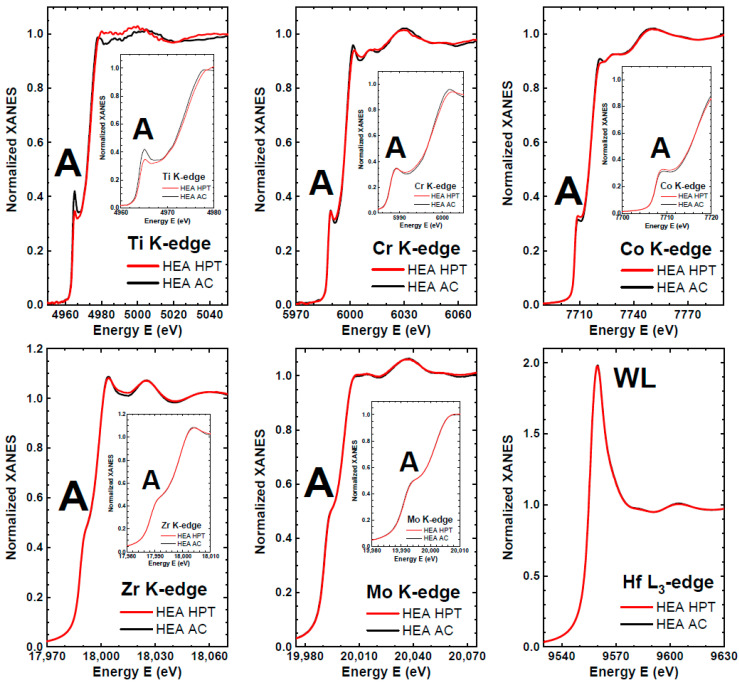
X-ray absorption near-edge spectra (XANES) of TiZrHfMoCrCo HEAs at the Ti, Cr, Co, Zr, and Mo K-edges and Hf L_3_-edge. The spectra for the as-cast (AC) sample and after high-pressure torsion (HPT) treatment are shown. The insets show an enlarged view of XANES spectra around the edge. The pre-edge A at the K-edges and the white line WL at the L_3_-edge are indicated.

The investigation of the local atomic structure in TiZrHfMoCrCo HEAs, both in the as-cast (AC) and HPT-treated states, was conducted via X-ray absorption spectroscopy (XAS) at the Ti, Cr, Co, Zr, and Mo K-edges and the Hf L3-edge. Figure 10 showcases the normalized X-ray absorption near-edge structure (XANES) spectra of these HEAs, with enlarged insets highlighting the region surrounding the edge. Generally, the XANES spectra display similarities at the Zr and Mo K-edges as well as the Hf L3-edge, exhibiting slight differences at the Cr and Co K-edges, and notably diverging at the Ti K-edge.

An observable decrease In shoulder A Intensity following HPT treatment at the Ti K-edge suggests an increase in local disorder or distortions around titanium atoms. Specifically, the K-edge XANES spectra reveal a distinctive pre-edge peak/shoulder A attributed to the 1s → nd (*n* = 3 for Ti, Cr, and Co; *n* = 4 for Zr and Mo) transition [48,49]. The increased natural line width of the 1s core level for heavier elements contributes to a less pronounced shoulder A at the Zr and Mo K-edges [50]. At the Hf L3-edge, a strong resonance immediately above the edge—referred to as the white line (WL)—arises due to the dipole-allowed transition 2p3/2(Hf) → 5d [51].

**Figure 11:**
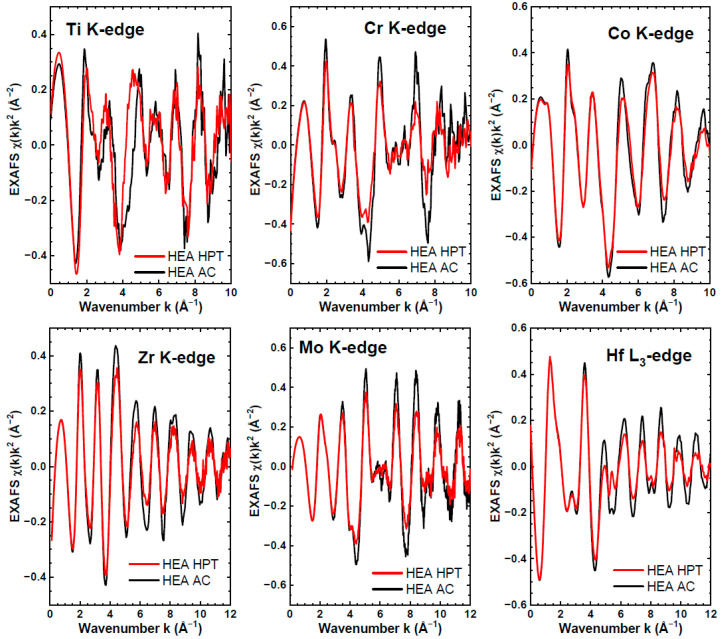
Extended X-ray absorption fine (EXAFS) spectra of TiZrHfMoCr HEAs at the Ti, Cr, Co, Zr, and Mo K-edges and Hf L_3_-edge. The spectra for the as-cast (AC) sample and after high-pressure torsion (HPT) treatment are shown.

The experimental EXAFS spectra, denoted as χ(*k*)*k*^2^ (where *k* represents the photo-electron wavenumber [20]), are depicted in Figure 11. It is noteworthy that the overall shape of the EXAFS spectra exhibits negligible changes following HPT treatment, indicating minimal alteration in the local environment surrounding the metal atoms, which remains largely intact. However, discernible damping of the EXAFS oscillations emerges, specifically at the Cr, Co, Zr, Mo, and Hf absorption edges, particularly at higher *k*-values. This attenuation results from an increase in static disorder, potentially stemming from a higher specific area of grain boundaries generated through strong grain refinement during the HPT process.

**Figure 12:**
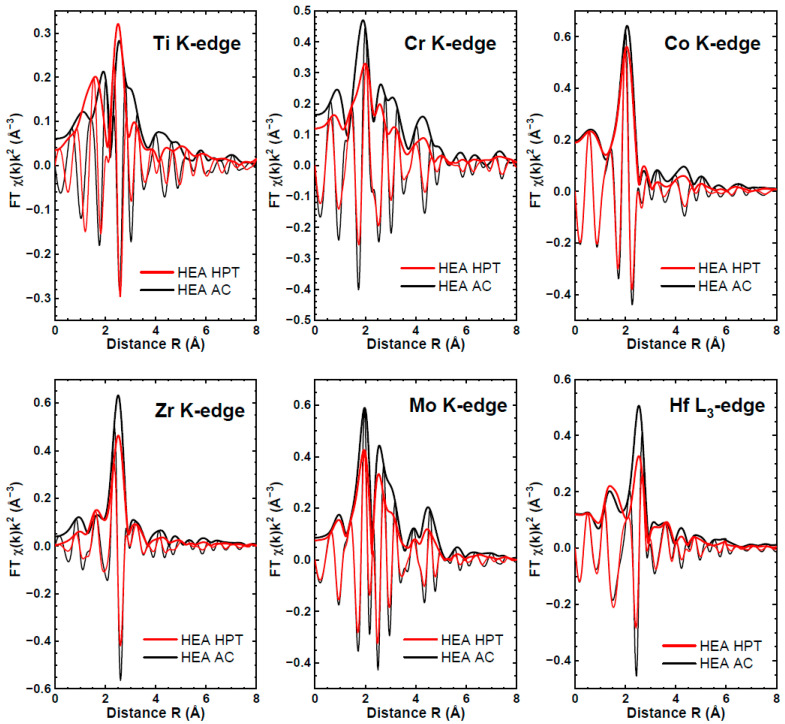
Fourier transforms (modulus and imaginary parts) of the Ti, Cr, Co, Zr, and Mo K-edges and Hf L3-edge EXAFS spectra of TiZrHfMoCr HEAs (as-cast (AC) and after high-pressure torsion (HPT) treatment).

**Figure 13:**
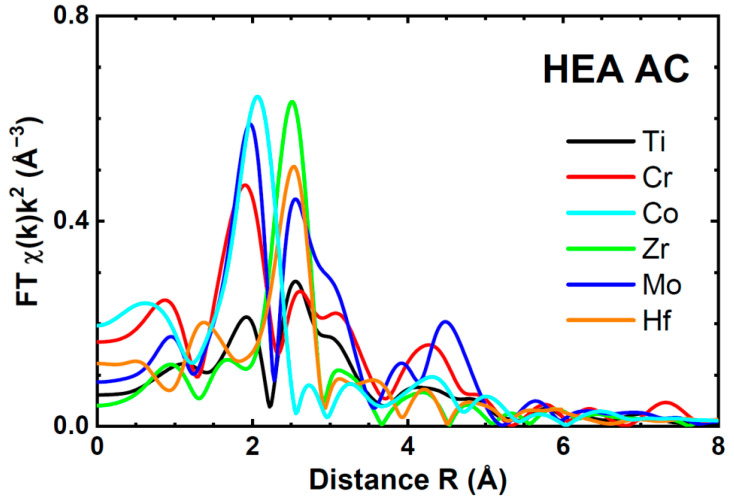
Comparison of the Fourier transform of the Ti, Cr, Co, Zr, and Mo K-edges and Hf L3-edge EXAFS spectra for as-cast (AC) TiZrHfMoCr HEA. Only the moduli of FTs are shown for clarity.

The Fourier transforms (FTs) derived from the EXAFS spectra of both the AC and HPT samples are showcased in Figure 12. Notably, the peaks within these FTs appear approximately 0.5 Å shifted to smaller distances relative to their crystallographic values, owing to the backscattering phase shift within the EXAFS formula [20]. The effects induced by high-pressure torsion manifest prominently around Cr, Zr, Mo, and Hf atoms, with discernible impacts observed on the local environment of Ti and Co as well. Qualitatively assessing the local environment within HEAs entails comparing their FTs across distinct absorption edges, as demonstrated in Figure 13 for the AC sample. The first peak in FTs represents the closest group of atoms, observed approximately 0.5 Å closer for Cr, Co, and Mo compared to Zr and Hf. Moreover, substantial distortion in the FT at the Ti K-edge indicates the likelihood of a disordered or multi-site environment surrounding Ti atoms. Hence, the HEAs likely encompass three distinctive local environments. Additionally, it is noteworthy that the position of the first peak in FTs correlates with the metallic radii size of the elements.

Analyzing the EXAFS spectra of HEAs presents a challenge owing to the multi-phase nature of the samples (as outlined in Table 1) and the diverse compositions of the coordination shells. The presence of varied atom types within the first coordination shell of a particular metal, occupying different crystallographic sites with varying degrees of distortion, complicates precise determinations of interatomic distances. Consequently, assessments typically yield estimations for these distances. Similar to our previous study [52], we approximated the effective radius of the first coordination shell of metal atoms by assessing the frequency of the EXAFS component governing the primary peak in the FTs. This estimation was executed through two simulation methods: the cumulant approach and the regularization technique [20]. Our findings indicate that the first shell radius approximately ranges from 2.45 to 2.7 Å for Cr, Co, and Mo, while spanning around 3.0 to 3.1 Å for Zr and Hf. However, titanium atoms inhabit a notably distorted environment characterized by a wide distribution of nearest atoms, spanning distances from 2.5 to 3.1 Å.

### 3.4. Mechanical Tests

#### 3.4.1. Microhardness Measurements

**Figure 14:**
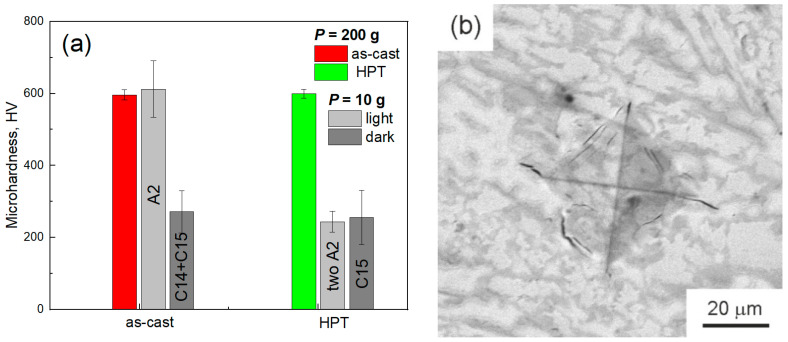
(**a**) The microhardness of alloy samples as-cast and after HPT processing. The total microhardness in the cast sample (red column) and after HPT (green column) was measured at a load of 200 g on the indenter, from the entire surface of the sample. The microhardness of individual phases was measured at a load of 10 g. (**b**) SEM image of the indenter imprint at a load of 200 g in the as-cast sample.

We conducted microhardness measurements on the alloy using two different indenter loads: 200 g and 10 g. At the 200 g load, the average diagonal size of the indentation was approximately 50 µm, while at 10 g, it was reduced to 4.5 µm. This load variation allowed us to estimate both the hardness of individual phases and the overall or “integral” hardness of the material, which is influenced not only by the properties of individual phases but also by the characteristics of grain boundaries. Figure 14a depicts the hardness values obtained at 200 g and 10 g loads for the alloy in its initial as-cast state and following HPT treatment. Surprisingly, despite load variations, the resulting hardness values were quite similar, both averaging around 600 ± 10 HV. Notably, when measuring the hardness of specific phases, such as bright and dark areas, a marked difference emerged after HPT treatment. Post-treatment, the hardness of bright areas significantly decreased by more than two-fold, dropping from 612 to 244 HV. In the initial state, the dark area consisted of a mixture of C14 and C15 phases, while the light area comprised the A2 phase. However, after HPT processing, the light area consisted primarily of the C15 phase, whereas the dark area comprised two distinct A2 phases.

Simultaneously, the hardness value remains relatively constant in dark areas. Evidently, in the initial alloy, the overall material hardness predominantly stems from the hardness of the light phase (A2). However, following HPT treatment, the material’s hardness seems to be influenced by the characteristics of the interphase grain boundary fractions. Assessing the comparison between light and dark areas in samples before and after HPT treatment is challenging due to the varying phase compositions in these areas. The obtained result is intricate and warrants further investigation to fully comprehend. The brittleness of the material is validated by the observation depicted in Figure 14b, illustrating the formation of cracks both under and around the indenter, underscoring the propensity of the material to undergo brittle fracture.

#### 3.4.2. Three-Point Bending

**Figure 15:**
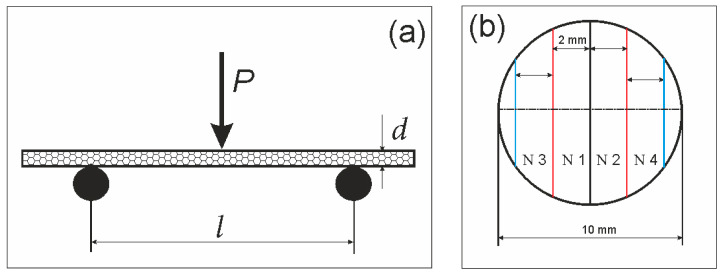
(**a**) Three-point bending test scheme and (**b**) sample cutting scheme for testing.

This study employed the three-point bending method (Figure 15a) for conducting mechanical tests, chosen due to its prevalent usage and relative simplicity, especially suitable for small-sized samples. All samples were shaped as disks measuring 10 mm in diameter and approximately 0.5 mm in thickness. They were specifically cut according to the schematic layout illustrated in Figure 15b for subsequent testing purposes.

**Figure 16:**
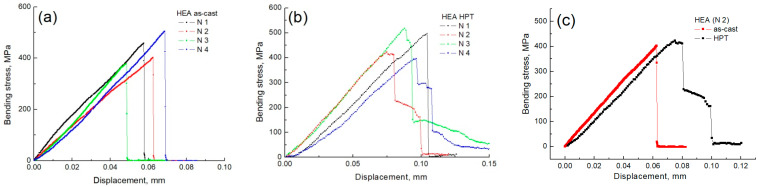
Loading curves of samples at three-point bending. The sample numbers N 1, N 2, N 3, and N 4 are shown in the diagram in Figure 15b. (**a**) In the initial state, (**b**) after HPT, (**c**) separately shows two samples with N 2 of two states, namely the initial as-cast sample (red line) and after HPT (black line).

According to the schematic presented in Figure 15b, two sets of samples were prepared—one from the initial as-cast HEA alloy and the other after undergoing HPT treatment. The first series, before HPT, featured samples with an average thickness of *d* = 0.63 ± 0.02 mm and an average width of *b* = 1.67 ± 0.02 mm. In contrast, the second series, post-HPT, consisted of samples from the same alloy, displaying dimensions of *d* = 0.53 ± 0.02 mm and *b* = 1.68 ± 0.02 mm. No additional treatments, whether thermal or mechanical, were applied to the samples. Based on the test outcomes, load–deflection (bending) dependencies were obtained (Figure 16). It is apparent from Figure 16a,b that HPT treatment had a negligible impact on the tensile strength of the sample. In the initial state (Figure 16a), the alloy showcased brittle fracture characteristics without exhibiting signs of ductility before failure; the sample fractures between deflections of 0.05 to 0.07 mm are shown in Figure 16a.

**Figure 17:**
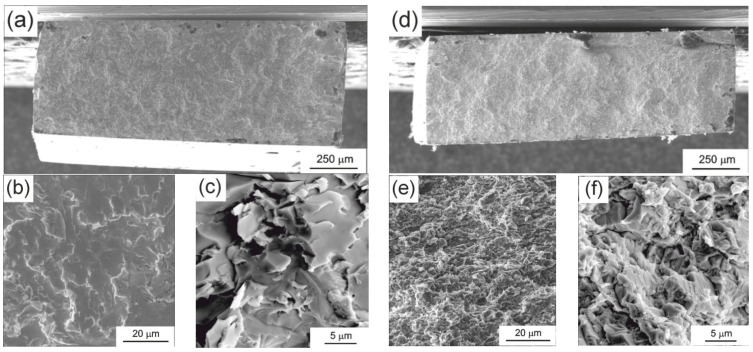
The fracture surface of the HEA samples N 2 after the tests: series 1 (**a**–**c**) for the initial as-cast alloy and series 2 (**d**–**f**) after HPT.

Following HPT treatment, the stress–displacement relationship (depicted in Figure 16b) showcases a notably distinct pattern. A yield plateau becomes evident at loads near the ultimate strength. Moreover, there is a marked increase in the maximum deflection of the samples before the onset of fracture—before HPT, the range was between 0.05 and 0.07 mm, whereas after HPT, it widened from 0.08 to 1.1 mm. Furthermore, the destruction of samples post-HPT treatment occurs in a stepwise manner. These findings collectively indicate that the HEA material exhibits enhanced plasticity after undergoing HPT. Additionally, the brittle nature of the fracture observed in the samples before HPT is corroborated by SEM micrographs of the fracture surface (Figure 17a–c). Notably, the fracture surface of samples pre-HPT appears flatter when compared to those post-HPT treatment (Figure 17d–f).

From the dependencies in Figure 16, the following three parameters for bending tests were calculated (see Table 3) using Equations (1)–(3):

(1) $$\mathrm{Bending}\;\mathrm{stress}\;\mathrm{for}\;\mathrm{rectangular}\;\mathrm{cross-section}\;\mathrm{samples}:\;\sigma =\frac{3Pl}{2bd^{2}}$$

(2) $$\mathrm{Modulus}\;\mathrm{of}\;\mathrm{elasticity}:\;E=\frac{l^{3}m}{4bd^{3}}$$

(3) $$\mathrm{Deformation}:\;\varepsilon =\frac{6Dd}{l^{2}}$$

where *l* is the distance between the supports (mm), *b* is the width of the sample (mm), *d* is the thickness of the sample (mm), *D* is the maximum deflection of the beam center (mm), *m* is the slope of the initial rectilinear section of the load–deflection curve (N/mm), and *v* is the loading speed 0.2 mm/min.

From the data provided in Table 3, it is evident that the average Young’s modulus value decreases after HDR processing compared to its initial state, albeit with a marginal alteration indicated by the presented error. The samples exhibit brittle fractures in their initial states (Figure 16a). However, after HPT processing, there is a transition in the fracture mode from brittle to brittle/plastic behavior (Figure 16b). This change in the fracture behavior may potentially contribute to the observed decrease in Young’s modulus.

The array of methodologies for manufacturing HEAs, akin to other metallic materials, is notably extensive. These encompass a spectrum of techniques like selective laser melting (SLM) [7,13], spark plasma sintering (SPS) [11], synthesis through magnetic levitation melting using a mixture of pure components [5], and vacuum arc melting [3], among others. For different manufacturing methods for HEAs’ yield varying microstructures, phase compositions, and mechanical properties, refer to [53] and Table 1 for a comprehensive overview. In our study, the alloy produced via levitation melting in an argon atmosphere [17] manifested three principal phases (Figure 1b). Contrastingly, in [3], the primary bcc phase was observed with trace amounts of Laves phases. While the phase composition in both our study and [3] remains consistent, differences emerge in the proportion of the formed phases and the microstructure of the HEA. Notably, our study demonstrates a smaller grain size. The TiZrHfMoCrCo alloy, developed by a group of researchers in [3], holds promise as a material capable of replacing medical alloys, such as SUS316L and Co–Cr–Mo, owing to its compatibility with bone tissue. Despite its commendable hardness, this alloy demonstrates brittleness. While this HEA remains a prospective candidate for implants, reducing its Young’s modulus could enhance its suitability for medical applications. Achieving this goal necessitates a deliberate selection of manufacturing methods and processing techniques to tailor the material’s properties accordingly.

In conclusion, we would like to reference another study conducted by our team on high-entropy alloys [54], which primarily investigated the influence of heat treatment on phase transformations within the alloy, alongside measurements of nanohardness and Young’s modulus. Given the considerable range observed in nanohardness values, our focus in this current article shifted towards examining microhardness and employing the three-point bending method. This article serves as a natural extension of the groundwork laid in [54], enriching our understanding of the alloy’s microstructure through the application of EBSD, TEM, and EXAFS techniques.

## 4. Conclusions

The investigation of the TiZrHfMoCrCo high-entropy alloy encompassed two distinct states: the initial as-cast state and its condition post-HPT treatment.

(1) The HPT treatment induced significant phase alterations and caused the disappearance of the hcp Laves phase C14 and the decomposition of the bcc phase A2 into two separate bcc A2 phases with distinct lattice parameters and compositions. One phase, enriched in hafnium, exhibited a lattice parameter of 3.445 nm, while the other, enriched in molybdenum, displayed a lattice parameter of 3.150 nm.
(2) Employing EBSD enabled the determination of the average grain size and the misorientation distribution of grain boundaries within each phase. The observation of the wetting of A2/A2 grain boundaries by layers of the C15 phase was also noted.
(3) The findings from the X-ray absorption spectroscopy analysis at the Ti, Cr, Co, Zr, and Mo K-edges, along with the Hf L3-edge, confirmed the retention of the local atomic structure of the metal atoms post-HPT treatment. A marginal increase in static disorder was detected, highlighting distinct local environments around Mo/Cr, Zr/Hf, and Ti atoms.
(4) Despite the fairly high microhardness value of 600 HV for the material, HPT exhibited a minimal impact on this property, although the microhardness of individual phases underwent significant changes, surpassing a two-fold difference.
(5) Pioneering tests were conducted and utilized the three-point bending method for the investigated alloy. The initial material exhibited greater brittleness compared to its post-HPT state. Additionally, the initial HEAs showcased a higher Young’s modulus of 135 GPa.

## Figures and Tables

**Table 1:** Phase and component composition of the TiZrHfMoCrCo sample and the corresponding types of phase crystal lattices.

Phase Designation	Phase Description, Spatial Group	Component Composition, wt.%	Type of Crystal Lattice
A2	BCC lattice (Ti,Zr,Hf)cub Im3m 4/m-32/m	Ti—13.5 ± 0.8 Cr—0.5 ± 0.5 Co—1.2 ± 0.9 Zr—28.4 ± 1.7 Mo—7.1 ± 1.7 Hf—46.4 ± 3.1	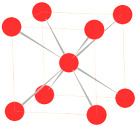
C15	The Laves phase	Ti—11.5 ± 0.6	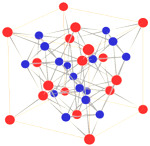
	FCC lattice	Cr—6.0 ± 0.6	
	Cr_2_Ti	Co—6.0 ± 0.6	
		Zr—13.8 ± 1.6	
	Fd3m	Mo—20.9 ± 2.4	
	F4_1/d-32/m	Hf—39.6 ± 3.0	
C14	The Laves phase	Ti—6.4 ± 0.7	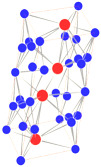
	HCP lattice	Cr—5.1 ± 0.6	
	Cr_2_Zr	Co—1.2 ± 0.8	
		Zr—11.3 ± 1.7	
	P63/mmc	Mo—34.9 ± 1.9	
	P6_3/m 2/m 2/c	Hf—41.3 ± 3.1	

**Table 2:** Chemical composition (wt.%) of the phases observed in the TiZrHfMoCrCo alloy after HPT, measured by SEM and TEM technologies.

Phases	Chemical Composition Measured by EDS/SEM	Chemical Composition Measured by EDS/TEM
A2 enriched in Hf, Zr	Ti—13.6 ± 0.5	Ti—12.9 ± 1.2
	Cr—1.0 ± 0.3	Cr—0.5 ± 0.1
	Co—1.6 ± 0.2	Co—1.0 ± 0.2
	Zr—25.8 ± 0.5	Zr—35.4 ± 5.1
	Mo—6.5 ± 0.3	Mo—6.5 ± 1.0
	Hf—51.5 ± 1.0	Hf—43.7 ± 5.5
A2 enriched in Mo	Ti—6.7 ± 0.3	Ti—5.0 ± 0.5
	Cr—5.4 ± 0.2	Cr—4.8 ± 0.7
	Co—1.3 ± 0.3	Co—1.0 ± 0.2
	Zr—10.6 ± 0.4	Zr—14.1 ± 2.0
	Mo—31.2 ± 0.6	Mo—38.3 ± 5.5
	Hf—44.8 ± 0.9	Hf—36.8 ± 4.6
C15	Ti—11.8 ± 0.5	Ti—9.5 ± 0.8
	Cr—5.3 ± 0.2	Cr—6.8 ± 1.0
	Co—7.1 ± 0.3	Co—5.1 ± 0.9
	Zr—15.4 ± 0.6	Zr—16.2 ± 2.2
	Mo—15.1 ± 0.6	Mo—24.0 ± 4.5
	Hf—45.3 ± 0.9	Hf—38.4 ± 4.6

**Table 3:** Calculated parameters from bending load curves.

	HEA (As-Cast)					HEA (HPT)				
	N 1	N 2	N 3	N 4	Average Value	N 1	N 2	N 3	N 4	Average Value
σ_max_ (MPa)	460	402	379	506	436 ± 28	498	420	519	396	458 ± 29
*E* (GPa)	135	97	113	111	114 ± 7	83	105	105	79	93 ± 7
ε (%)	0.3	0.4	0.3	0.4	0.35 ± 0.05	0.6	0.4	0.4	0.5	0.48 ± 0.09

## Data Availability

The data presented in this study are available upon request from the corresponding author.
